# Cell polarity, cell adhesion, and spermatogenesis: role of cytoskeletons

**DOI:** 10.12688/f1000research.11421.1

**Published:** 2017-08-25

**Authors:** Linxi Li, Ying Gao, Haiqi Chen, Tito Jesus, Elizabeth Tang, Nan Li, Qingquan Lian, Ren-shan Ge, C. Yan Cheng

**Affiliations:** 1 The Mary M. Wohlford Laboratory for Male Contraceptive Research, Center for Biomedical Research, New York, USA; 2 The Second Affiliated Hospital & Yuying Children’s Hospital, Wenzhou Medical University, Wenzhou, Zhejiang, China

**Keywords:** spermatogenesis, cytoskeletons, Cell polarity, spermiation, sertoli cell, testis, testes

## Abstract

In the rat testis, studies have shown that cell polarity, in particular spermatid polarity, to support spermatogenesis is conferred by the coordinated efforts of the Par-, Crumbs-, and Scribble-based polarity complexes in the seminiferous epithelium. Furthermore, planar cell polarity (PCP) is conferred by PCP proteins such as Van Gogh-like 2 (Vangl2) in the testis. On the other hand, cell junctions at the Sertoli cell–spermatid (steps 8–19) interface are exclusively supported by adhesion protein complexes (for example, α6β1-integrin-laminin-α3,β3,γ3 and nectin-3-afadin) at the actin-rich apical ectoplasmic specialization (ES) since the apical ES is the only anchoring device in step 8–19 spermatids. For cell junctions at the Sertoli cell–cell interface, they are supported by adhesion complexes at the actin-based basal ES (for example, N-cadherin-β-catenin and nectin-2-afadin), tight junction (occludin-ZO-1 and claudin 11-ZO-1), and gap junction (connexin 43-plakophilin-2) and also intermediate filament-based desmosome (for example, desmoglein-2-desmocollin-2). In short, the testis-specific actin-rich anchoring device known as ES is crucial to support spermatid and Sertoli cell adhesion. Accumulating evidence has shown that the Par-, Crumbs-, and Scribble-based polarity complexes and the PCP Vangl2 are working in concert with actin- or microtubule-based cytoskeletons (or both) and these polarity (or PCP) protein complexes exert their effects through changes in the organization of the cytoskeletal elements across the seminiferous epithelium of adult rat testes. As such, there is an intimate relationship between cell polarity, cell adhesion, and cytoskeletal function in the testis. Herein, we critically evaluate these recent findings based on studies on different animal models. We also suggest some crucial future studies to be performed.

## Introduction

**Figure 1.**
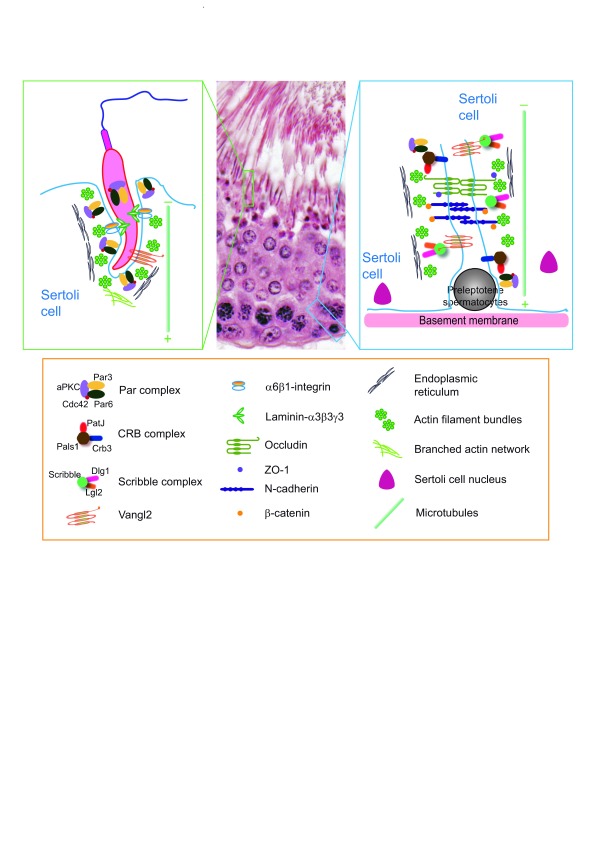
Distribution of the three polarity protein complexes (or modules) and the Van Gogh-like 2 (Vangl2)-based complex in the seminiferous epithelium of adult rat testes. As discussed in the text and based on recently published findings, the Par-based and the Vangl2-based complexes are the only corresponding polarity protein and planar cell polarity (PCP) protein complexes found at or near the Sertoli cell–spermatid interface known as the apical ectoplasmic specialization (ES). However, all three polarity protein complexes and the Vangl2-based PCP complex are detected at the basal ES/blood-testis barrier. Studies have shown that all three polarity protein complexes and the Vangl2-based PCP protein complex exert their regulatory effects through the F-actin–based cytoskeleton by modulating the spatiotemporal expression of actin regulatory proteins (for example, Eps8, Apr3, and palladin) at the ES. Studies have also shown that the Vangl2-based complex is structurally linked to the Scribble ^16^. Since the polarized microtubules (MTs)—as noted by the presence of the fast (+, plus) growing end versus the slow (−, minus) growing end—are intimately related to the actin microfilaments at the ES and since studies based on the three animal models have shown that the MT-based cytoskeleton is also involved in changes at the ES integrity induced by adjudin, F5-peptide, or formin 1 expression level, it is likely that MT is also one of the regulatory targets of the polarity protein complexes and Vangl2-based PCP protein complex. This possibility requires additional investigation in future studies.

Spermatogenesis takes place in the seminiferous tubule, the functional unit of the testis. It is known that each pair of testes in mice and rats produces about 10 ^1^ and 70 ^2^ million spermatozoa per day in comparison with men, who produce about 200 million spermatozoa per day ^2, 3^ throughout their entire adult life. Thus, it is conceivable that there are enormous cellular activities in the seminiferous epithelium to meet this substantial output. It is noted that developing germ cells are aligned in an orderly fashion in the seminiferous epithelium so that they can be nurtured by Sertoli cells throughout their development without disruption. Studies have shown that germ cells, in particular developing spermatids, are highly polarized cells in the testis. For instance, spermatid heads from steps 8–19 in the rat (or steps 8–16 in the mouse) testis align almost perpendicularly toward the basement membrane whereas the tails point toward the tubule lumen. This alignment ensures that the maximal number of developing elongating/elongated spermatids are packed in an orderly fashion in the limited space of the seminiferous epithelium to support this sperm output ^4– 6^. On the other hand, Sertoli cells are also highly polarized cells in the seminiferous epithelium. For instance, tight junction (TJ) and basal ectoplasmic specialization (ES) are restrictively localized toward the base of the Sertoli cell, closest to the basement membrane; and nucleus, Golgi apparatus, and lysosomes are conspicuously located toward the base of the Sertoli cell ( Figure 1). This morphological layout is also important to support spermatogenesis so that pertinent physiological and cellular events can take place in an orderly manner in the cellular compartments. Studies in the last decade have shown that the three polarity complexes that confer apico-basal polarity found in *Drosophila*, *Caenorhabditis elegans* and other mammalian cells ^7– 10^ are also present in the testis as they are also necessary to support spermatid and Sertoli polarity as well as their adhesive function, including the Par- ^11– 13^, Scribble- ^14^, and Crumbs-based ^15^ polarity complexes. Furthermore, many of the partner proteins that are known to support the physiologically important functions of these three polarity protein complexes ^4, 7, 8^ also are found in the *Drosophila* testis ^11, 14, 15^. Recent studies have shown that, besides these cell polarity proteins, planar cell polarity (PCP) proteins, such as Vangl2 (Van Gogh-like 2), the mammalian homolog of Van Gogh, Vang, protein in *Drosophila*, are also found in the testis ^16^. Unlike cell polarity proteins, PCP proteins are crucial to confer convergent extension during embryogenesis such as gastrulation so that tissue narrows (that is, convergent) along one axis concomitant with elongation (extension) along a perpendicular axis as the result of polarized cell migration to generate antero-posterial axis ^17– 19^. In adult tissues, the most obvious PCP is seen in hair cells of the cochlear and cuticular hair cells of the insect as well as in developing spermatids, ensuring alignment of a field of polarized cells within the plane of the epithelium ^20– 22^.

Recent studies using several animal models have unequivocally demonstrated the significance of cell polarity proteins and also PCP proteins in spermatogenesis. Interestingly, these studies using different animal models have shown that, at least in developing spermatids, the ultrastructure that plays a crucial role to confer cell polarity and PCP is the testis-specific actin-rich adherens junction called ES ^23– 28^. The ES is found either at the Sertoli cell–spermatid interface (steps 8–19 in the rat testis or steps 8–16 in the mouse testis), known as the apical ES, or at the Sertoli cell–cell interface called the basal ES ( Figure 1). Furthermore, disruption of either cell polarity or PCP protein function in the testis is often associated with a disruption of spermatid adhesion, leading to early release of spermatids from the seminiferous epithelium. Thus, cell polarity and PCP proteins are critical components of spermatid adhesion in the testis. More importantly, a disruption of either cell polarity or PCP protein function is often associated with a disruption of both actin- and microtubule (MT)-based cytoskeletal organization. These findings illustrate that cell polarity or PCP function is intimately related to the two cytoskeletal elements in the testis. In this review, we critically evaluate these recent findings and this information should provide a better understanding of spermatid development during spermatogenesis and may provide better approaches for non-hormonal male contraceptive development. Such data will also provide insightful information to assist in the management of some currently unexplained cases of male infertility.

## Role of cell polarity in Sertoli and spermatid adhesion during spermatogenesis

Studies using different animal models have shown that changes in spermatid polarity often are associated with subsequent disruption of spermatid adhesion because of subtle changes in cytoskeletal organization in the seminiferous epithelium ^25, 29– 32^. In several of these animal models, these observations have demonstrated unequivocally that spermatid polarity is supported by (i) the actin filament bundles at the Sertoli cell–elongating/elongated spermatid interface known as the apical ES and (ii) the adjacent MT network near the apical ES. Disruption of elongating/elongated spermatid polarity is the result of apical ES degeneration (or its malfunction) and disorganization of the supporting actin microfilaments. This leads to spermatid exfoliation, which is limited to elongating/elongated spermatids that reside at or near the tubule lumen, regardless of the stages of the epithelial cycle. This is manifested by the release of elongating/elongated spermatids analogous to spermiation in stages other than stage VIII tubules. However, if the track-like structures conferred by MTs are also disrupted, elongating/elongated spermatids without functional apical ES are trapped deep inside the epithelium, and step 19 spermatids are persistently detected in the seminiferous epithelium in stage IX–XIV tubules until they are phagocytosed and lysed by the Sertoli cells. These findings thus support the notion that spermatid polarity is intimately related to the functional status and organization of F-actin or MTs (or both) in the seminiferous epithelium.

For Sertoli cells, cell adhesion function can be readily assessed and quantified by monitoring the TJ-permeability barrier across the Sertoli cell epithelium by using cells cultured on Matrigel-coated bicameral units, as earlier described ^33– 36^. Studies that monitor the Sertoli cell–TJ barrier function have shown that the use of a loss-of-function approach by RNA interference (RNAi) has demonstrated unequivocally that all three polarity proteins play a role in conferring the Sertoli cell–TJ barrier function. Furthermore, the cell polarity complexes (that is, the Par-, Scribble-, and Crumbs-based complexes) and the PCP protein complex (for example, Vangl2/Prickle complex) have recently been shown to modulate the F-actin- or MT-based (or both) cytoskeletal function ^4, 5, 11, 14, 15^. For most of the discussion on cell polarity, we focus our attention on the cell polarity proteins since there is only a single report on PCP protein Vangl2 in the literature ^16^. Herein, we evaluate these findings as follows.

### Adjudin model

Adjudin, 1-(2,4-dichlorobenzyl)-1H-indazole-3-carbohydrazide, is an indazole-based chemical synthesized in the late 1990s and shown to be a non-hormonal and reversible male contraceptive in rats ^37– 39^ and rabbits ^40^ following its administration by oral gavage. Adjudin exerts its anti-fertility effects by disrupting the apical ES initially which takes just ~6–8 hr to occur, to be followed by a disruption of the desmosome and gap junction which takes ~3–6 days to be seen. This causes extensive germ cell exfoliation, and virtually all tubules are eventually devoid of germ cells within about 1–2 weeks ^31, 41, 42^. Since the population of undifferentiated spermatogonia and Sertoli cells is not notably affected, spermatogenesis rebounds and germ cells of all classes gradually re-populate the entire seminiferous epithelium with a return of fertility ^43, 44^. Because of these observations, rats treated with a single dose of adjudin at 50 mg/kg body weight (b.w.) by inducing germ cell exfoliation, most notably elongating/elongated spermatids ^42^, have been used to study the biology of spermiation by identifying the proteins, genes, and signaling molecules and signaling pathways pertinent to the regulation of spermatid adhesion and de-adhesion ^45– 51^. However, it was noted that, within about 6–9 hours following a single dose of adjudin treatment, there was extensive disruption of spermatid polarity wherein spermatid heads no longer pointed to the basement membrane perpendicularly but deviated by as much as 90° from the normal orientation and often by 180° ^11, 31, 52^, illustrating that spermatid polarity is grossly affected. These changes usually occur at the time spermatids begin their detachment from the Sertoli cell in the seminiferous epithelium ^11^. In fact, studies have shown that adjudin perturbs the distribution of polarity proteins Par6 ^11^ and Scribble ^14^ in the seminiferous epithelium of adult rat testes following treatment with a single dose of adjudin (50 mg/kg b.w.) administered by oral gavage. For instance, within 12 hours of treatment with adjudin, Scribble is no longer highly expressed at the blood-testis barrier (BTB) in the epithelium ^14^. Scribble is considerably diminished at the BTB by 2 days, and by 4 days, the expression of Scribble was virtually non-detectable at the BTB ^14^. On the other hand, Par6 is expressed predominantly at the basal ES/BTB but also at the apical ES and co-localizes with occludin at the BTB, and nectin-3 is also expressed at the apical ES ^11^. Treatment of rats with adjudin for 12 hours considerably downregulated Par6 expression at the basal ES/BTB but upregulated Par3 expression at the apical ES, and Par6 is no longer tightly associated with the elongated spermatid heads but appeared as aggregates that moved away from the apical ES site ^11^. In this context, it is of interest to note that a knockdown of Par3, Par6, or Crb3 by RNAi using corresponding specific small interfering RNA (siRNA) duplexes perturbs the Sertoli cell–TJ barrier function, making it “leaky” because of an internalization of either TJ or basal ES proteins at the Sertoli cell–cell interface ^11, 15^, thereby failing to support the barrier function. In contrast, a triple knockdown of Scribble and its partner proteins Dlg1 (Discs large 1) and Lgl2 (Lethal giant larvae 2) by RNAi promotes the Sertoli cell–TJ barrier function, making it “tighter” ^14^. Furthermore, triple knockdown of Scribble, Dlg1, and Lgl2 by RNAi in the testis *in vivo* promotes the Sertoli cell–TJ barrier function by recruiting more occludin to the basal ES/BTB through an increase in F-actin distribution at the site ^14^. Collectively, these findings illustrate that the Par- and Crb3-based polarity complexes promote the Sertoli cell–TJ barrier function by making it “tighter” in normal testes but that Scribble promotes the Sertoli cell–TJ barrier by making it “leaky” in normal testes. In short, the Par/Crb3- and the Scribble-based complexes serve as molecular switches to make the TJ barrier either “tight” or “leaky”. This notion is in agreement with the function of these three polarity complexes in other mammalian cells or tissues (or both) in which the Par- and Crumbs-based polarity complexes are usually working in concert but their localization or function or both are mutually exclusive (that is, antagonizing) with the Scribble-based polarity complex to control apico-basal polarity ^4, 7, 8^. Nonetheless, the adjudin-mediated downregulation on the steady-state protein levels of Par6 and its associated proteins such as JAM-C ^11^, and also Scribble and its partner proteins Dlg1 and Lgl2 ^14^, compromises the intriguing spatiotemporal functional relationship between these three polarity complexes in the seminiferous epithelium to support cell polarity function and spermatogenesis. This adjudin-induced loss of polarity proteins in the testis also perturbs the ability of Sertoli cells to support spermatid polarity through the only anchoring device at the Sertoli cell–spermatid interface (from step 8 to step 19 spermatids in the rat testis) called apical ES. It is noted that the most remarkable feature of the apical ES is the array of actin microfilament bundles found in the Sertoli cell that are sandwiched in between the cisternae of endoplasmic reticulum and the apposing Sertoli–spermatid plasma membranes ( Figure 1). Thus, it is logical to speculate that spermatid polarity is dependent mostly on the F-actin–based cytoskeleton, which is likely the regulatory target of the cell polarity proteins. In fact, findings from several studies have supported this concept. First, a knockdown of Par3 or Par6 by RNAi using specific siRNA duplexes versus non-targeting negative control duplexes in Sertoli cells has shown that a loss of either Par3 or Par6 leads to mis-localization of both TJ proteins (for example, JAM-A and ZO-1) and basal ES proteins (for example, N-cadherin and α-catenin), which are known to use F-actin for attachment ^11^, supporting the notion that the F-actin organization has been disrupted. Second, a knockdown of Crb3 by RNAi in Sertoli cells also grossly perturbs the organization of actin microfilaments across the cell cytosol, wherein actin microfilaments no longer stretch across the entire cell but become extensively truncated ^15^. Furthermore, Crb3 knockdown Sertoli cells are shown to have considerable reduction in their capability to induce actin bundling based on a biochemical assay ^15^. In contrast, a triple Scribble/Dlg1/Lgl2 knockdown in a Sertoli cell epithelium promotes re-organization of F-actin at the cell cortical zone to better support tighter TJ barrier function ^14^. Taken collectively, these findings demonstrate unequivocally that one of the targets by which polarity protein complexes exert their regulatory function is the cytoskeleton. Perhaps this is mediated by recruiting actin or MT regulatory proteins (or both) to the corresponding cytoskeletal network to modify its organization through the binding partners of the cell polarity complex. Consistent with these findings, a recent study by silencing PCP protein Vangl2 in Sertoli cells using RNAi also impeded the organization of F-actin across the Sertoli cell cytosol wherein actin microfilaments were grossly truncated, and the ability of the Vangl2-silenced Sertoli cells to induce actin filament bundles was also considerably perturbed ^16^. Thus, the PCP protein Vangl2/Prickle complex also exerts its regulatory effects through changes in F-actin–based cytoskeletal organization.

### F5-peptide and NC1 peptide models

In adult rat testes, cellular events that take place across the seminiferous epithelium during the epithelial cycle are known to be coordinated by a local functional axis known as the apical ES-BTB-basement membrane axis ^53, 54^. This axis functionally connects different major structural components across the epithelium to coordinate cellular events involving autocrine-based factors generated locally. For instance, during the release of sperm transformed from step 19 spermatids at spermiation at stage VIII of the epithelial cycle, a surge in matrix metalloprotease 2 (MMP-2) expression at the apical ES facilitates the cleavage of the apical ES adhesion protein complex α6β1-integrin/laminin-α3β3γ3 ^55– 58^. This generates biologically active fragments of laminin chains ^59^ (such as F5-peptide, a 50–amino acid peptide derived from domain IV of laminin-γ3 chain ^60^), which are capable of inducing further apical ES breakdown to promote its degeneration in order to facilitate spermiation ^61^. More importantly, studies have shown that these biologically active fragments such as F5-peptide are also capable of inducing remodeling of the Sertoli cell BTB near the basement membrane both *in vitro* and *in vivo* ^59– 61^. Additionally, these biologically active peptides are able to modulate the basement membrane function by downregulating the expression of β1-integrin, a component of hemidesmosome at the Sertoli cell–basement membrane interface ^59^. In the testis, the basement membrane is a modified form of extracellular matrix ^62, 63^, and the observation that biologically active peptides derived from the apical ES could modulate the basement membrane function prompted us to examine whether a manipulation of β1-integrin, a hemidesmosome protein, could modify the BTB function. Indeed, a knockdown of β1-integrin in Sertoli cells cultured *in vitro* with an established TJ-permeability barrier by more than 80% by RNAi was found to considerably downregulate the steady-state level of TJ adaptor protein ZO-1 ^59^. This impeded the localization of TJ-integral membrane protein occludin and basal ES-integral membrane protein N-cadherin, failing to support the BTB function ^59^. These findings are physiologically significant since they illustrate the presence of a local functional loop between the BTB and the basement membrane to modulate local cellular events essential to spermatogenesis. Subsequent studies have also demonstrated that non-collagenous 1 (NC1) domain released from collagen α3 (IV) chain ^64^, likely via the action of MMP-9 which is highly expressed in the basal region of the seminiferous epithelium ^65^, in the basement membrane is capable of modifying BTB dynamics ^64^. It is through this local regulatory axis, involving locally produced and biologically active peptides such as the F5-peptide and the NC1 domain peptide, that the three ultrastructures across the seminiferous epithelium, namely the apical ES, BTB/basal ES, and basement membrane/hemidesmosome, are functionally connected and coordinated. This supports the cellular events of spermiation and BTB remodeling that take place concurrently but at opposite ends of the seminiferous epithelium. Interestingly, it was noted that overexpression of F5-peptide by transfecting testes *in vivo* with a mammalian expression vector pCI-neo containing the full-length F5-peptide cDNA (or through direct administration of the synthetic F5-peptide into the testis), this led to extensive germ cell exfoliation besides reversible disruption of the Sertoli cell BTB integrity ^60, 61^. Thus, besides perturbing the apical and basal ES function, the F5-peptide is capable of disrupting desmosome, the major anchoring junction between step 1–7 spermatids/spermatocytes and Sertoli cells. More importantly, following overexpression of F5-peptide in the testis, many elongating/elongated spermatids displayed defects of polarity since these spermatids had their heads pointing more than 90° away from instead of aligning perpendicular to the basement membrane, and some spermatid heads even pointed toward the tubule lumen ^60^. Since the transfection efficiency based on the use of a Polyplus *in vivo*-jetPEI as a transfection medium was estimated to be about 60–70%, which is almost twice as high when compared with a conventional transfection medium, the number of tubules that were shown to be affected was also about 70% in the study ^61^. This loss of spermatid polarity eventually led to germ cell exfoliation, and up to about 70% of the tubules were found to be devoid of spermatids, illustrating that the F5-peptide has the potential to become a male contraceptive peptide if the transfection efficacy can be further improved. Nonetheless, F5-peptide can be used to improve the bioavailability of adjudin when it can be co-delivered with adjudin using a multidrug nanoparticle-based approach ^66^. More importantly, F5-peptide was found to induce extensive disruption of the F-actin- and MT-based cytoskeletons in the seminiferous epithelium of rat testes following its overexpression *in vivo*. Most notably, the track-like structures conspicuously conferred by MTs and also some by F-actin that laid perpendicularly against the basement membrane, and stretched across the seminiferous epithelium were largely disrupted in the affected tubules ^61^. These track-like structures were either broken, considerably diminished or laid at right angles to the longitudinal axis of the tubule. This illustrates that the tracks that supported spermatid transport (and other organelle transport such as residual bodies, phagosomes, and endocytic vesicles) in the Sertoli cell were grossly perturbed. As such, even though the anchoring device—the apical ES—was grossly disrupted, many elongated spermatids were embedded deeply inside the seminiferous epithelium, even in stage IX, X, XI, XII, and XIII tubules ^61^. They failed to be emptied into the tubule lumen versus other elongated spermatids because there were no tracks to support their transport to the tubule lumen for spermiation. These spermatids were eventually phagocytosed and eliminated by Sertoli cells ^61^. Furthermore, many multinucleated round spermatids were noted in affected tubules, and these multinucleated spermatids were surrounded by MTs, destined to be degenerated via lysosomal degradation ^61^. This observation confirms the disruption of intracellular trafficking events due to a failure in endocytic vesicle transport mechanisms, resulting from the loss of the MT- and actin-based track-like structures as noted in other mammalian cells or tissues ^67– 70^. In short, the MT- and F-actin–based cytoskeletons as the target of the F5-peptide and their disruption leads to changes in spermatid polarity and adhesive function, causing either their eventual premature release from the epithelium or their failure to be transported across the epithelium to support spermatogenesis. These findings also illustrate that the cytoskeleton networks, which connect the infrastructures of cell junctions across the epithelium and which also support multiple cellular events along the length of the epithelium, are the target to the F5-peptide. However, it remains to be investigated whether the F5-peptide is working in concert with the Par-, Crumbs-, Scribble-, or Vangl2-based complexes to modulate spermatid polarity and adhesion through changes in the cytoskeletal function in the testis.

### Formin 1 model

Formin 1, a 180-kDa polypeptide, is a member of the formin family of proteins which are known to nucleate actin, resulting in the development of long stretches of actin microfilaments rapidly, and also to stabilize MTs ^71– 75^. A functional formin 1 nucleator is a dimeric protein, besides serving as the major actin nucleator that promotes formation of linear actin filament from the barbed end (also the fast growing plus (+) end) of an existing filament, is also capable of bundling actin filaments through its F-actin bundling domain ^76, 77^. This makes it also an actin bundler by organizing actin filaments into bundles such as those found at the apical and basal ES. More importantly, each formin 1 polypeptide chain contains an MT-binding domain (MTB) near its N-terminal region ^74, 76^, which is known to play a role in stabilizing MTs in mammalian cells. Since the F-actin- and MT-based networks are closely localized to support apical and basal ES function ^23, 78, 79^, the predominant presence of formin 1 at the basal ES/BTB in stage VI–VII, but not VIII, tubules ^80^ thus supports the notion that formin 1 is nucleating actin microfilaments while being involved in the assembly of actin filament bundles and the stabilization of the MT network at the ES in stage VI–VII tubules. The absence of formin 1 at both the apical and basal ES sites in the seminiferous epithelium at stage VIII of the epithelial cycle ^80^ is also necessary to support the release of sperm at spermiation and the transport of preleptotene spermatocytes across the BTB in stage VIII tubules by downregulating actin nucleation processes at both sites. Furthermore, formin 1 in stage VI-VII tubules co-localizes with MTs ^81^, confirming the notion that formin 1 is crucial to MT dynamics besides its role as a nucleator of actin microfilaments. In the testis, a knockdown of formin 1 in the testis *in vivo* by RNAi was found to induce premature release of spermatids from the epithelium in stage VI, VII, and early VIII tubules ^80, 81^. This is likely due to the loss of the ability of the apical ES to maintain its adhesion function because of a failure in actin nucleation activity conferred by formin 1. However, in many stage IX tubules, numerous elongated spermatids remained embedded deep inside the epithelium, and many of these step 19 spermatids had defects in polarity as noted by their heads pointed toward the tubule lumen, at least 90° deviated from the basement membrane ^80, 81^. Furthermore, phagosomes that should have been transported to the base of the tubules for their eventual degradation via the lysosomal pathway in stage VIII tubules ^82^ as noted in control testes were found to be conspicuously present in the epithelium near the tubule lumen in stage IX-X tubules following the knockdown of formin 1 in the testis ^80, 81^. Subsequent studies have shown that such defects, namely the loss of spermatid polarity and defects on spermatid adhesion and spermatid/organelle transport, were the result of disruptive changes in the organization of F-actin and MTs. In brief, the track-like structures conferred by F-actin and also MTs were considerably diminished following formin 1 knockdown in the testis ^81^, so that spermatids, even in the absence of functional apical ES to support spermatid adhesion, failed to be transported to the tubule lumen to undergo spermiation due to the lack of track-like structures to sustain their support. These findings also demonstrate unequivocally that spermatid polarity is a cytoskeleton-dependent cellular function, supported by both the actin- and MT-based cytoskeletons. It remains to be investigated whether formin 1 knockdown would perturb the spatiotemporal expression of the Par-, Crumbs-, or Scribble-based polarity complexes (or a combination of these) or the Vangl2-based PCP protein complex.

## Concluding remarks and future perspectives

As briefly reviewed herein, cell polarity is intimately related to the underlying actin- and MT-based cytoskeletons that support cell adhesion protein complexes and the three cell polarity modules plus the Vangl2-based PCP protein complex. However, except for the adjudin model, changes in the spatiotemporal expression of the three cell polarity complexes have yet to be examined, even though there are obvious defects in spermatid polarity in both animal models. Furthermore, it is now known that the PCP protein complex Vang/Prickle exerts its effects to modulate F-actin organization by inhibiting polymerization of actin microfilaments ^83– 85^, and Vangl2 is also known to be highly expressed at the basal ES/BTB and localized also in the track-like structures conferred by either F-actin- or MT-based cytoskeleton in the seminiferous epithelium of adult rat testes ^16^. However, it remains to be examined whether the Vangl2/Prickle signaling pathway is involved in the three animal models, contributing to the phenotypes summarized herein. Much work is needed in the next few years to precisely identify the mechanism(s) and also the participating molecules by which polarity complexes or PCP protein complexes (or both) exert their effects to modulate the organization of F-actin and MTs in the testis.
